# Supplementary material for: Do Tonkean macaques (Macaca tonkeana) perceive what conspecifics do and do not see?
Source: PeerJ. 2016 Feb 22;4:e1693. doi: 10.7717/peerj.1693 (PMC4768696; doi:10.7717/peerj.1693)
Supplement: Supplemental Information 2 — Summary of all statistical models presented in the paper. [file peerj-04-1693-s002.docx]

Summary of GLMM1 based on binary data concerning the first direction towards food or no food adopted by subordinates in the four experimental conditions. Fixed effects: Condition; Trial number; Hierarchical rank difference. Random effect: Dominant. Number of data = 435; Number of subjects = 11; Number of dyads = 21.

|  | Df | AIC | LRT | Pr(Chi) |
| --- | --- | --- | --- | --- |
| <none> |  | 391.94 |  |  |
| Condition | 3 | 564.28 | 178.342 | < 2.2e-16*** |
| Number of trial | 1 | 397.74 | 7.799 | 0.005227** |
| Hierarchical rank difference | 1 | 393.15 | 3.215 | 0.072985 |

Summary of multiple comparisons between the four experimental conditions with Tukey corrections from GLMM1.

| comparisons | Estimate | | Std.Error | | z value | Pr(>\|z\|) | |
| --- | --- | --- | --- | --- | --- | --- | --- |
| 2 - 1 == 0 | | 1.9594 | | 0.5064 | 3.869 | | 0.000563 *** |
| 3 - 1 == 0 | | -2.8905 | | 0.3876 | -7.458 | | < 1e-04 *** |
| 4 - 1 == 0 | | -1.5330 | | 0.3446 | -4.449 | | < 1e-04 *** |
| 3 - 2 == 0 | | -4.8499 | | 0.5333 | -9.094 | | < 1e-04 *** |
| 4 - 2 == 0 | | -3.4924 | | 0.4912 | -7.111 | | < 1e-04 *** |
| 4 - 3 == 0 | | 1.3575 | | 0.3386 | 4.010 | | 0.000310 *** |

Summary of GLMM2 based on binary data concerning the first direction towards food or no food adopted by subordinates in conditions 1 and 2. Fixed effects: Condition; Trial number; Hierarchical rank difference. Random effect: Dyad. Number of data = 225; Number of subjects = 11; Number of dyads = 20.

|  | Df | AIC | LRT | Pr(Chi) |
| --- | --- | --- | --- | --- |
| <none> |  | 140.01 |  |  |
| Condition | 1 | 151.28 | 13.2690 | 0.0002698 *** |
| Number of trial | 1 | 141.37 | 3.3665 | 0.0665352 |
| Hierarchical rank difference | 1 | 143.03 | 5.0219 | 0.0250291 * |

Summary of comparisons between conditions 1 and 2 from GLMM2.

|  | Estimate | StdError | Zvalue | Pr(>\|z\|) |
| --- | --- | --- | --- | --- |
| (Intercept) | 2.45562 | 0.90592 | 2.711 | 0.006715 ** |
| Condition 2 | 1.88561 | 0.56674 | 3.327 | 0.000878 *** |
| Number of trial | 0.03262 | 0.01893 | 1.723 | 0.084810 |
| Hierarchical rank difference | -0.27573 | 0.12178 | -2.264 | 0.023568 * |

Summary of GLMM3 based on binary data concerning the first direction towards hidden food or visible food adopted by subordinates in conditions 1 and 2. Fixed effects: Condition; Trial number; Hierarchical rank difference. Random effect: Dyad. Number of data = 225; Number of subjects = 11; Number of dyads = 20.

|  | Df | AIC | LRT | Pr(Chi) |
| --- | --- | --- | --- | --- |
| <none> |  | 140.01 |  |  |
| Condition | 1 | 151.28 | 13.2690 | 0.0002698 *** |
| Number of trial | 1 | 141.37 | 3.3665 | 0.0665352 |
| Hierarchical rank difference | 1 | 143.03 | 5.0219 | 0.0250291 * |

Summary of comparisons between conditions 1 and 2 from GLMM3.

| Fixed effects |  |  |  |  |  |
| --- | --- | --- | --- | --- | --- |
|  | | Estimate | Std.Error | z value | Pr(>\|z\|) |
| (Intercept) | | 2.45562 | 0.90592 | 2.711 | 0.006715 ** |
| Condition 2 | | 1.88561 | 0.56674 | 3.327 | 0.000878 *** |
| Number of trial | | 0.03262 | 0.01893 | 1.723 | 0.084810 |
| Hierarchical rank difference | | -0.27573 | 0.12178 | -2.264 | 0.023568 * |

Summary of GLM4 based on binary data concerning the first direction towards hidden food or visible food adopted by dominants in conditions 3 and 4. Fixed effects: Condition; Trial number; Hierarchical rank difference. Number of data = 210; Number of subjects = 11; Number of dyads = 20.

|  | Df | Deviance | AIC | LRT | Pr(>Chi) |
| --- | --- | --- | --- | --- | --- |
| <none> |  | 274.09 | 282.09 |  |  |
| Condition | 1 | 275.88 | 281.88 | 1.7948 | 0.18034 |
| Number of trial | 1 | 277.51 | 283.51 | 3.4234 | 0.06428 |
| Hierarchical rank difference | 1 | 274.36 | 280.36 | 0.2702 | 0.60318 |

Summary of comparisons between conditions 3 and 4 from GLM4.

|  | Estimate | Std Error | z value | Pr(>\|z\|) |
| --- | --- | --- | --- | --- |
| (Intercept) | 0.236197 | 0.345135 | 0.684 | 0.4937 |
| Condition 4 | -0.387539 | 0.290438 | -1.334 | 0.1821 |
| Number of trial | 0.014891 | 0.008245 | 1.806 | 0.0709 |
| Hierarchical rank difference | 0.023981 | 0.046292 | 0.518 | 0.6044 |

Summary of GLMM5 based on binary data concerning the outcome 1 in the four experimental conditions. Fixed effects: Condition; Trial number; Hierarchical rank difference. Random effect: Dominant. Number of data = 196; Number of subjects = 11; Number of dyads = 20.

|  | Df | AIC | LRT | Pr(Chi) |
| --- | --- | --- | --- | --- |
| <none> |  | 260.06 |  |  |
| Condition | 3 | 596.20 | 342.13 | < 2e-16 *** |
| Number of trial | 1 | 259.66 | 1.59 | 0.20693 |
| Hierarchical rank difference | 1 | 261.58 | 3.52 | 0.06077 |

Summary of multiple comparisons between the four experimental conditions with Tukey corrections from GLMM5.

| Comparisons | Estimate | Std. Error | z value | Pr(>\|z\|) |
| --- | --- | --- | --- | --- |
| 2 - 1 == 0 | 0.4066 | 0.6030 | 0.674 | 0.9028 |
| 3 - 1 == 0 | 6.5355 | 0.7599 | 8.600 | <0.001 *** |
| 4 - 1 == 0 | 5.3938 | 0.6731 | 8.013 | <0.001 *** |
| 3 - 2 == 0 | 6.1288 | 0.6974 | 8.788 | <0.001 *** |
| 4 - 2 == 0 | 4.9872 | 0.6030 | 8.271 | <0.001 *** |
| 4 - 3 == 0 | -1.1416 | 0.4751 | -2.403 | 0.0728 |

Summary of GLMM6 based on binary data concerning the outcome 2 in conditions 1 and 2. Fixed effects: Condition; Trial number; Hierarchical rank difference. Random effect: Dominant. Number of data = 30; Number of subjects = 7; Number of dyads = 9.

|  | Df | AIC | LRT | Pr(Chi) |
| --- | --- | --- | --- | --- |
| <none> |  | 160.76 |  |  |
| Condition | 1 | 182.12 | 23.3616 | 1.342e-06 *** |
| Number of trial | 1 | 166.38 | 7.6203 | 0.005772 ** |
| Hierarchical rank difference | 1 | 159.81 | 1.0486 | 0.305838 |

Summary of comparisons between conditions 1 and 2 from GLMM6.

| Fixed Effects | Estimate | Std.Error | z value | Pr(>\|z\|) |
| --- | --- | --- | --- | --- |
| (Intercept) | -5.26133 | 1.14044 | -4.613 | 3.96e-06 *** |
| Condition 2 | 2.43110 | 0.60512 | 4.018 | 5.88e-05 *** |
| Number of trial | 0.03936 | 0.01492 | 2.637 | 0.00836 ** |
| Hierarchical rank difference | 0.10667 | 0.11188 | 0.953 | 0.34037 |

Summary of GLMM7 based on binary data concerning the outcome 3 in conditions 1 and 2. Fixed effects: Condition; Trial number; Hierarchical rank difference. Random effect: Dominant. Number of data = 177; Number of subjects = 11; Number of dyads = 19.

|  | Df | AIC | LRT | Pr(Chi) |
| --- | --- | --- | --- | --- |
| <none> |  | 214.88 |  |  |
| Condition | 1 | 233.41 | 20.5253 | 5.885e-06 *** |
| Number of trial | 1 | 218.37 | 5.4862 | 0.01917 * |
| Hierarchical rank difference | 1 | 218.37 | 5.4890 | 0.01914 * |

Summary of comparisons between conditions 1 and 2 from GLMM7.

| Fixed Effects | Estimate | Std. Error | z value | Pr(>\|z\|) |
| --- | --- | --- | --- | --- |
| (Intercept) | 3.80333 | 0.70262 | 5.413 | 6.20e-08 *** |
| Condition 2 | -1.77949 | 0.43122 | -4.127 | 3.68e-05 *** |
| Number of trial | -0.02851 | 0.01277 | -2.234 | 0.0255 * |
| Hierarchical rank difference | -0.16468 | 0.07207 | -2.285 | 0.0223 * |

Summary of GLMM8 based on binary data concerning the outcome 4 in conditions 3 and 4. Fixed effects: Condition; Trial number; Hierarchical rank difference. Random effect: Dominant. Number of data = 25; Number of subjects = 6; Number of dyads = 6.

|  | Df | AIC | LRT | Pr(Chi) |
| --- | --- | --- | --- | --- |
| <none> |  | 139.79 |  |  |
| Condition | 1 | 143.31 | 5.5120 | 0.01889 * |
| Number of trial | 1 | 139.62 | 1.8203 | 0.17728 |
| Hierarchical rank difference | 1 | 139.08 | 1.2849 | 0.25699 |

Summary of comparisons between conditions 3 and 4 from GLMM8.

| Fixed Effects | Estimate | Std.Error | z value | Pr(>\|z\|) |
| --- | --- | --- | --- | --- |
| (Intercept) | -3.76122 | 1.32996 | -2.828 | 0.00468 ** |
| Condition 4 | 1.16705 | 0.52319 | 2.231 | 0.02570 * |
| Number of trial | 0.01889 | 0.01415 | 1.335 | 0.18185 |
| Hierarchical rank difference | -0.15539 | 0.13744 | -1.131 | 0.25821 |

Summary of GLM9 based on binary data concerning the link between first direction adopted by subordinates and outcome 2 in conditions 1 and 2. Fixed effect: First direction adopted by subordinates. Number of data = 30; Number of subjects = 7; Number of dyads = 9.

|  | Df | Deviance | AIC | LRT | Pr(>Chi) |
| --- | --- | --- | --- | --- | --- |
| <none> |  | 111.87 | 115.87 |  |  |
| First direction adopted by subordinate | 1 | 168.43 | 170.43 | 56.563 | 5.443e-14 *** |

Summary of GLM9.

|  | Estimate | Std.Error | z value | Pr(>\|z\|) |
| --- | --- | --- | --- | --- |
| (Intercept) | 0.3023 | 0.3198 | 0.945 | 0.345 |
| First direction adopted by subordinate | -3.3737 | 0.5017 | -6.724 | 1.77e-11 *** |
