# Supplementary material for: Do Tonkean macaques (Macaca tonkeana) perceive what conspecifics do and do not see?
Source: PeerJ. 2016 Feb 22;4:e1693. doi: 10.7717/peerj.1693 (PMC4768696; doi:10.7717/peerj.1693)
Supplement: Supplemental Information 3 — Descriptive statistics of first direction adopted by subordinates and dominants and outcome of the tests in the four experimental conditions. [file peerj-04-1693-s003.docx]

|  |  | Condition 1 | Condition 2 | Condition 3 | Condition 4 |
| --- | --- | --- | --- | --- | --- |
| Subordinate | Food (hidden) | 74% | 67% | 2% | 9% |
|  | Food (visible) | 6% | 27% | 21% | 39% |
|  | None | 20% | 6% | 77% | 52% |
| Subordinate | Hidden | 92,5% | 71,79% | 8,70% | 18,75% |
|  | Visible | 7,5% | 28,21% | 91,30% | 81,25% |
| Dominant | Food (hidden) | 1% | 0% | 66% | 58% |
|  | Food (visible) | 96% | 79% | 34% | 42% |
|  | None | 3% | 21% | 0% | 0% |
| Dominant | Hidden | 1,03% | 0% | 66% | 58% |
|  | Visible | 98,97% | 100% | 34% | 42% |
|  | Outcome 1 | 5,0% | 8,8% | 92,0% | 80,9% |
|  | Outcome 2 | 4,0% | 21,6% | 0,0% | 0,9% |
|  | Outcome 3 | 90,0% | 69,6% | 0,0% | 1,8% |
|  | Outcome 4 | 1,0% | 0,0% | 8,0% | 16,4% |
|  | Number of data | 100 | 125 | 100 | 110 |
|  | Number of subject tested | 10 | 11 | 10 | 11 |
|  | Number of dyad tested | 12 | 20 | 16 | 20 |
